# Multivessel Spontaneous Coronary Artery Dissection: Amberen as a Possible Risk Factor

**DOI:** 10.7759/cureus.4191

**Published:** 2019-03-06

**Authors:** Zeid Nesheiwat, Muhammad A Mangi, FNU Zafrullah, Daniel Kosinski

**Affiliations:** 1 Cardiology, University of Toledo Medical Center, Toledo, USA; 2 Internal Medicine, Steward Carney Hospital, Tufts University School of Medicine, Boston, USA

**Keywords:** spontaneous coronary artery dissection, scad, amberen, myocardial infarction, multivessel spontaneous coronary artery dissection

## Abstract

Spontaneous coronary artery dissection (SCAD) is a rare phenomenon that causes acute, life-threatening myocardial infarction. Most notably occurring in the female population, certain risk factors have been implicated in SCAD including pregnancy, hormone therapy, stimulant drug use, connective tissue disorders and systemic inflammatory disorders. However, the effects of over-the-counter supplements have not been widely studied in SCAD. We have a case of acute multivessel type 2 SCAD involving the obtuse marginal artery and posterior descending artery that may have been secondary to Amberen, an over-the-counter supplement used to relieve symptoms of menopause. This is a rare case of multivessel SCAD possibly caused by an over-the-counter supplement not previously known to trigger this disease process.

## Introduction

Spontaneous coronary artery dissection (SCAD) is described as a non-thrombotic and non-atherosclerotic cause of myocardial infarction. It is caused when there becomes a disruption within the coronary wall leading to the development of a false lumen that then impairs blood flow distally [1]. The actual prevalence of SCAD is difficult to quantify because, in some cases, it appears similar to atherosclerotic disease (Type 3 SCAD) by coronary angiography [2]. It is speculated that approximately 0.5% of myocardial infarctions are a result of SCAD. However, the prevalence of SCAD becomes increasing more common in younger females who experience myocardial infarction with some reports indicating a prevalence of up to 25% [3]. Risk factors for this disease include female sex, pregnancy, connective tissue disorders (i.e., Ehlers-Danlos, Fibromuscular Dysplasia, Marfan syndrome), systemic inflammatory disorders and estrogen containing medications [4]. Some reports have noted SCAD occurring with stressors such as physical exertion or emotional exertion. Now, with the development of advanced imaging such as optical coherence tomography and intravascular ultrasound, the prevalence of SCAD can be further established. The gold standard for diagnosis is coronary angiography and the mainstream treatment, in most cases, is goal-directed medical therapy since the vessels are typically too fragile for percutaneous coronary intervention (PCI) therapy [5]. Here, we report a 55-year-old female presented with a chief complaint of chest pressure since starting Amberen. Cardiac catheterization revealed SCAD involving the terminal branches of obtuse marginal (OM) and posterior descending artery (PDA). The patient was started on aggressive medical management and sent home with early outpatient clinic follow-up.

## Case presentation

**Figure 1:**
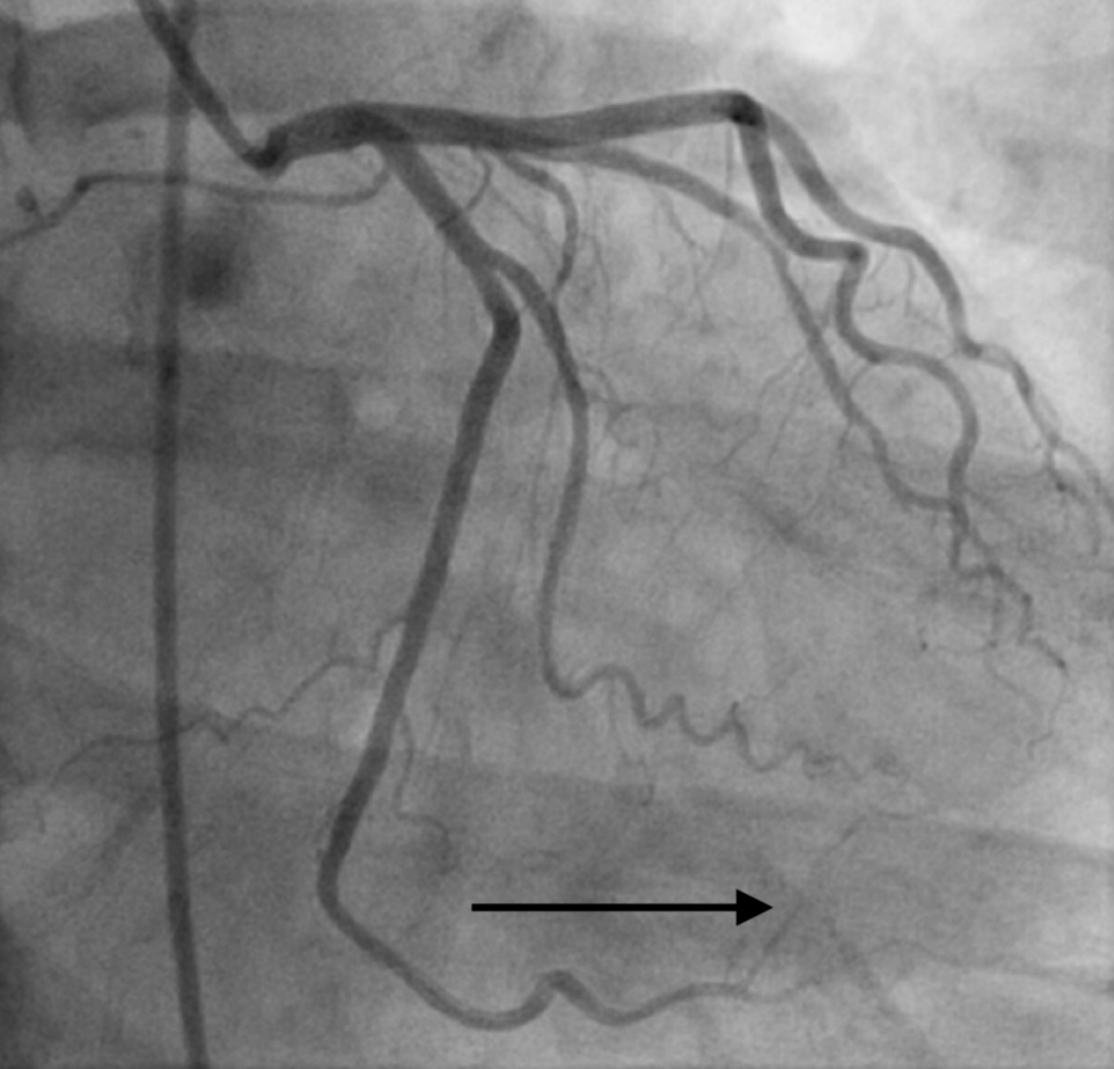
Type II spontaneous coronary artery dissection (SCAD) involving the proximal branch of the obtuse marginal artery.

**Figure 2:**
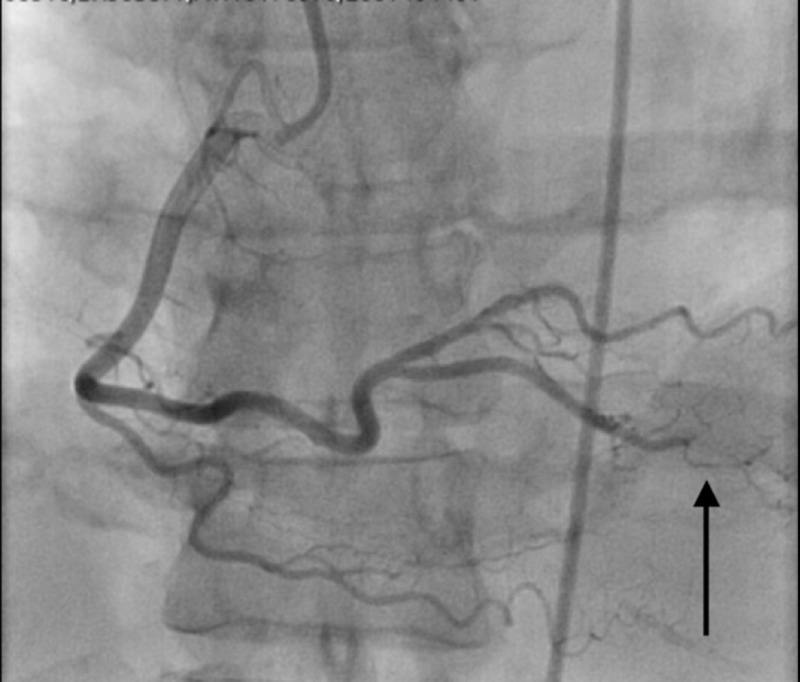
Type II spontaneous coronary artery dissection (SCAD) involving the terminal branch of the posterior descending artery.

**Figure 3:**
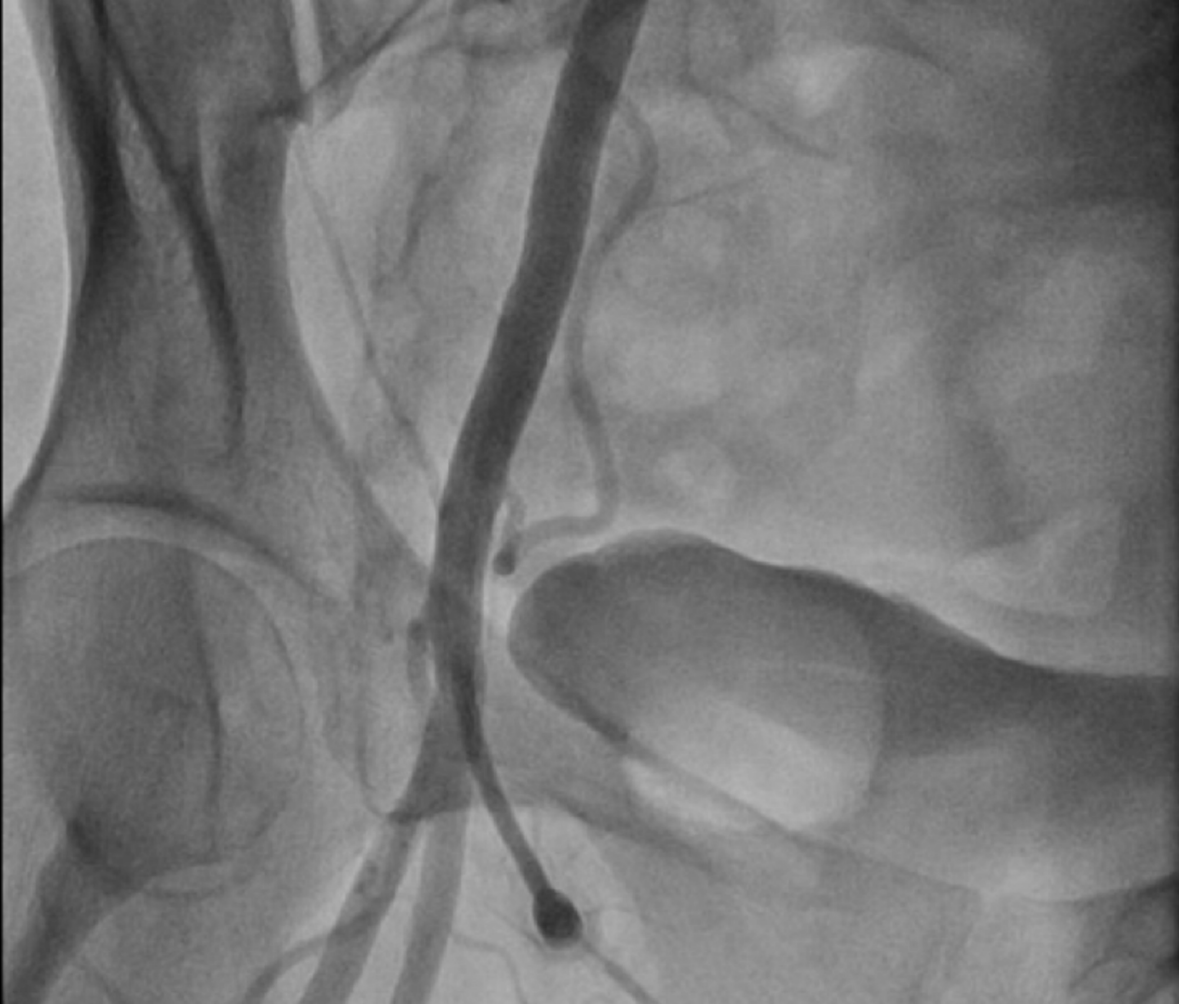
Femoral angiogram showed no evidence of fibromuscular dysplasia.

A 55-year-old female with a past medical history of hypothyroidism presented to the emergency department with complaint of chest pressure that radiated to her neck and jaw. The patient endorsed intermittent chest pressure, diaphoresis, nausea, and dizziness for the last two weeks. On examination, the patient was hemodynamically stable on room air. Initial electrocardiogram revealed normal sinus rhythm without evidence of ischemic changes, but initial troponin was elevated at 1.94 ng/mL. Chest X-ray was unremarkable. The patient was immediately given aspirin 325 mg, started on heparin infusion, and cardiology was consulted. Goal-directed medical therapy was started for acute coronary syndrome (ACS). The following day, cardiac catheterization was performed which revealed multivessel coronary artery disease with the appearance of spontaneous coronary artery dissection involving the terminal branch of OM and PDA (Figures 1-2). Both of these vessels were of small caliber and not amendable to PCI. In addition, there was no evidence of fibromuscular dysplasia on femoral angiogram (Figure 3). Left ventriculogram (LV gram) showed an ejection fraction of 55-60% with evidence of akinesis of the mid-inferior segment of the left ventricle. The patient was medically managed and troponin decreased during her hospitalization. Due to the patient’s history of miscarriages and intermittent loose stool, thorough workup was performed to evaluate for hypercoagulable disorders including, but not limited to, antiphospholipid antibody and celiac disease, which all came back negative. After conducting further investigation, it was brought to our attention that the patient had been taking an over-the-counter supplement, called Amberen, to treat her symptoms of menopause. She stated that this medication was just started recently and her chest symptoms correlated with the start of the supplement. The patient had an uneventful recovery and was discharged home with strict follow-up in the outpatient cardiology clinic.

## Discussion

Despite the advancement of medical technology and research, spontaneous coronary artery dissection still remains a deadly threat to the cardiovascular system. With an in-hospital mortality rate of 4.2% and a recurrent in-hospital myocardial infarction rate of 4.6%, emergency recognition and treatment are imperative [1,6]. Risk factors include female sex, pregnancy, connective tissue disorders, and estrogen containing medications [4].

Multivessel SCAD occurs in 9% to 23% of cases with the involvement of the OM being reported in 15% to 40% of cases and PDA at less than 10% of cases [4,2]. It is also important to note that SCAD is characterized in three types depending on the radiographic findings on angiography. Type 1 SCAD is when a false lumen is identified within the affected vessel. Type 2 SCAD, the most common form, is when the affected vessel shows diffuse narrowing. Type 3 SCAD resembles atherosclerotic disease and its true prevalence is unknown [7]. The mainstream treatment is goal-directed medical therapy and strict risk modification. PCI is typically not performed due to vessel fragility, but some reports have shown success [8]. It is important to note that prognosis for SCAD is varied, with a long-term adverse event rate of 16.8% and, of those, 10.4% being a recurrent of SCAD [6]. To aid in the prevention of recurrent episodes, it is advised for patients to limit the amount of exertional activities [9]. Strict follow-up in the outpatient clinic and medication compliance is heavily indicated in this population.

In this particular case, a patient who had been taking a supplement not presumed as a risk factor developed SCAD. Amberen, to date, has not previously been known to induce SCAD, opening up many questions relating to its mechanism and safety. Was Amberen the cause of this patient’s SCAD? Does Amberen contain estrogen-related compounds since it is used for menopause symptom relief? These questions are still a matter of debate, but this case report strongly suggests Amberen-induced SCAD. Moreover, additional research is needed to evaluate this supplement in order to answer these crucial questions.

## Conclusions

Spontaneous coronary artery dissection is a devastating diagnosis. Healthcare professionals need to be aware of the risk factors, diagnosis, treatment, and prognosis of this disease. In this case, we presented a female patient who developed multivessel SCAD of the OM and PDA possibly secondary to Amberen, an over-the-counter supplement used to help relieve symptoms of menopause. Although Amberen use is not an established risk factor for SCAD, this case report is aimed to show that additional research needs to be done in an effort to identify further risk factors. Every healthcare provider should be well-educated regarding SCAD since it may present in any patient despite risk factors, like in this case report.
